# Development of a reporter feline herpesvirus-1 for antiviral screening assays

**DOI:** 10.1186/s13567-024-01430-7

**Published:** 2024-12-18

**Authors:** Jia Yang, Li Li, Fuqiang Xu, Fan Jia

**Affiliations:** 1 Shenzhen Key Laboratory of Viral Vectors for Biomedicine, Shenzhen-Hong Kong Institute of Brain Science, Shenzhen Institute of Advanced Technology, Chinese Academy of Sciences, Shenzhen, 518055 China; 2 Key Laboratory of Quality Control Technology for Virus-Based Therapeutics, the Brain Cognition and Brain Disease Institute, Shenzhen Institute of Advanced Technology, Chinese Academy of Sciences, Shenzhen, 518055 China; 3 University of Chinese Academy of Sciences, Beijing, 100049 China; 4 Faculty of Life and Health Sciences, Shenzhen University of Advanced Technology, Shenzhen, 518107 China

**Keywords:** Feline herpesvirus type 1, reporter virus, luciferase, fluorescent protein, antiviral drug screening

## Abstract

Feline herpesvirus type 1 (FHV-1), a member of the *Herpesviridae* family, is one of the most important pathogens that causes upper respiratory tract disease in felines. Following infection, FHV-1 can spread retrogradely to the trigeminal ganglia, establishing a life-long latency. Although vaccines are available for routine feline vaccination, FHV-1 is still an agent that poses a serious threat to feline health. There are currently no specific drugs for the treatment of FHV-1. To facilitate the screening of antiviral drugs, we constructed a reporter FHV-1 virus, which expresses a secreted Gaussia luciferase (GLuc) and a bright green fluorescent protein, mNeonGreen. The reporter virus shows slower growth than does the wild-type FHV-1. The expression of the two reporter genes, Gluc and mNeonGreen, was consistent with viral propagation and remained stable during continuous passage in CRFK cells, even after twenty rounds. In addition, the known inhibitor ganciclovir was used to confirm the characteristics of the reporter virus for drug screening. We found that the reporter FHV-1 is suitable for antiviral screening assays. Overall, our work provides a useful tool for screening drugs to combat FHV-1.

## Introduction

Feline herpesvirus-1 (FHV-1) is one of the most important agents that can cause serious upper respiratory infections and ocular infections in felines, and it is present in the feline population worldwide [1]. FHV-1 spreads mainly between felines through direct contact with infected cats, and it is shed through ocular, nasal, and oral secretions [1]. Vaccines are currently available for routine feline vaccination, and they include modified live and inactivated virus vaccines [2]. Importantly, these vaccines can reduce the severity of the disease, but they cannot prevent infection, virus shedding, virus latency in the trigeminal ganglion, or reactivation [1, 3]. Furthermore, gene-deleted live virus vaccines have been constructed and evaluated for their safety and efficacy in combating FHV-1 in vivo [4–7]. However, specific and efficient drugs are urgently needed for treating FHV-1 disease.

To discover drugs to treat FHV-1, many antiviral drugs that were developed to treat human diseases caused by closely related human herpesviruses, such as herpes simplex virus type 1, have been used directly to treat felines infected by FHV-1. However, this translational drug use might be inefficient or harmful to felines. For example, acyclovir is especially efficient for clinically treating humans infected with HSV-1, but it is less efficient against FHV-1 than against HSV-1 [8–10]. On the basis of this evidence, the efficiency and safety of all drugs approved for use against human herpesviruses should be tested before they can be used to treat feline disease. However, the efficiency of many drugs and compounds, such as acyclovir, adefovir, bromovinyldeoxyuridine, cidofovir, ganciclovir, idoxuridine, penciclovir, foscarnet, trifluridine and vidarabine, against FHV-1 is currently being tested [10]. A traditional method for evaluating the inhibitory ability of a drug is the plaque reduction neutralization test (PRNT). This method is classic and effective, although it is time consuming and laborious. Therefore, rapid, sensitive, and convenient tools for screening antiviral drugs are needed. To date, many reporter viruses have been developed by engineering a specific reporter gene into the viral genome, such as herpes simplex virus [11–13], pseudorabies virus [14–16], varicella zoster virus [17], human cytomegalovirus [18], Japanese encephalitis virus [19], and yellow fever virus [20], which can be applied in many aspects of the biomedical field, such as in discovering antiviral drugs and assessing their efficacy against viruses. However, there is still no established reporter FHV-1 for antiviral drug screening.

On the basis of the requirements for a rapid, sensitive, and convenient antiviral screening assay, the bright green fluorescent protein mNeonGreen and the secreted Gaussia luciferase (GLuc) were selected to generate a double-reporter FHV-1. mNeonGreen is a product of the cephalochordate *Branchiostoma lanceolatum* [21], and it provides a direct method for assessing the ability of drugs to inhibit viruses through fluorescence visualization. GLuc can be spontaneously released from cells into culture media, and it has a greater signal strength than other luciferases [22, 23]. Its activity can be measured directly in culture media without cell lysis. Therefore, the combination of mNeonGreen and GLuc should provide a rapid, sensitive, and convenient tool for screening drugs to combat FHV-1.

## Materials and methods

### Cells, viruses and reagents

Crandell-Rees feline kidney (CRFK) cells were maintained in Dulbecco's modified Eagle medium (DMEM, L110KJ, Basal Media, China) supplemented with 10% foetal bovine serum (FBS, 10,091,148, Gibco, New Zealand) and incubated at 37 °C in 5% CO_2_. The wild-type FHV-1 (feline herpesvirus 1 strain GD2019, GenBank accession no. PP942287) and the double-reporter FHV-1 were added to CRFK cells cultured in DMEM containing 2% FBS at 37 °C in 5% CO_2_. The monoclonal antibody against mNeonGreen was purchased from Proteintech (32F6, USA). The goat anti-rabbit secondary antibody was purchased from Abcam (ab205718, USA). The drug ganciclovir was purchased from Aladdin (G407871, China). DMSO was purchased from Sigma‒Aldrich (D2650, USA). The CCK-8 kit was purchased from Vazyme (A311-02, China). A Gaussian luciferase flash assay kit was purchased from Pierce (16,159, USA).

### Generation of a double reporter FHV-1

**Figure 1:**
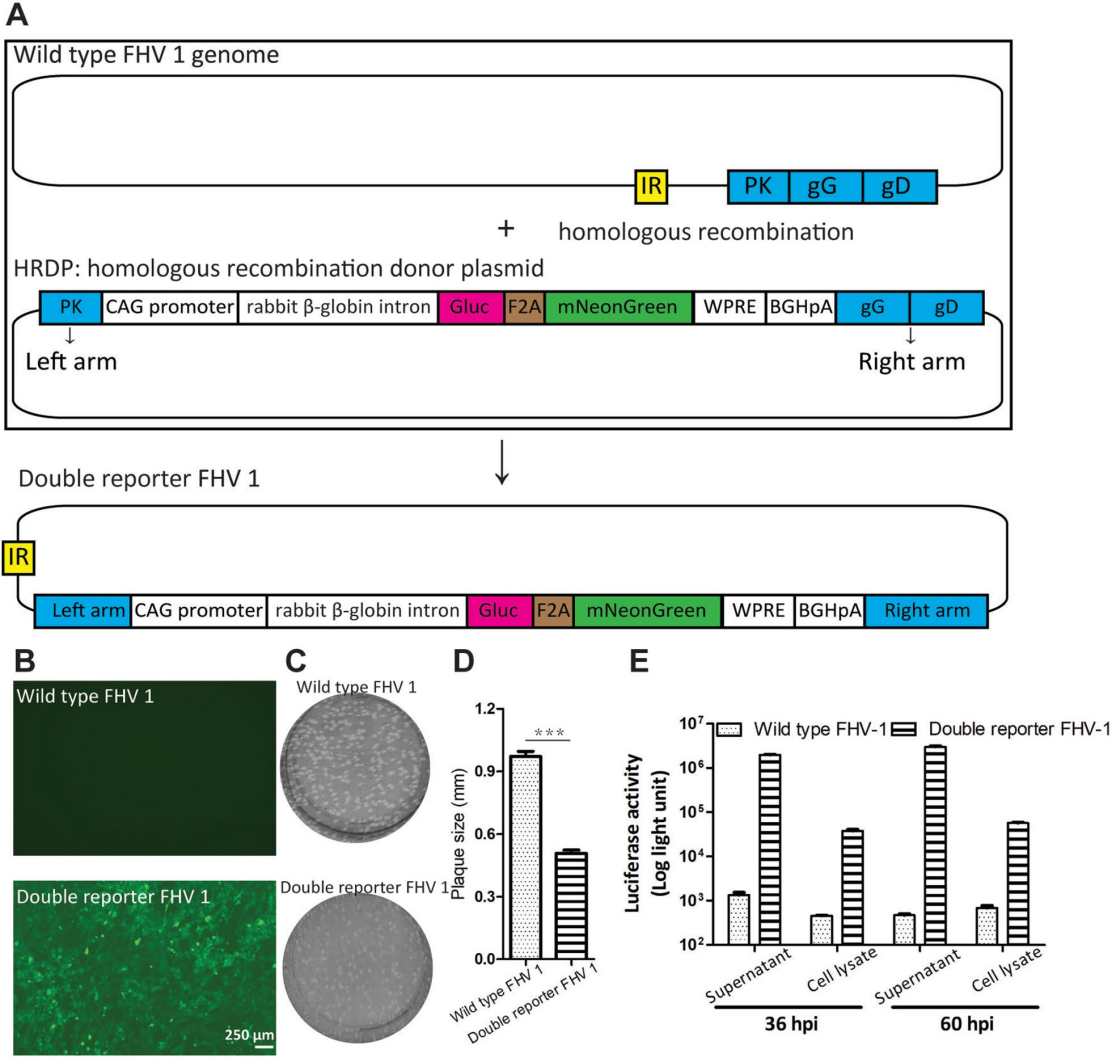
**Generation of a double-reporter FHV-1.** **A** Schematic of the double-reporter FHV-1. The mNeonGreen-F2A-Gluc expression cassette was inserted into the middle of the gG of FHV-1 by homologous recombination between the homologous recombination donor plasmid (HRDP) and the FHV-1 genome with the help of the left and right arms. **B** Analysis of mNeonGreen expression in CRFK cells infected with wild-type FHV-1 and the double reporter FHV-1. The CRFK cells were infected with wild-type FHV-1 and the double reporter FHV-1 at an MOI of 0.05, and mNeonGreen was observed with a fluorescence microscope at 60 hpi. **C** Plaque size of the wild-type FHV-1 and the double-reporter FHV-1. At 60 hpi, the supernatants of these two viruses were collected and used for plaque assays. **D** Compared with the wild-type FHV-1, the FHV-1 double reporter has a smaller plaque size. Thirty plaques of the double-reporter FHV-1 and wild-type strains were randomly selected for testing plaque diameter. Statistical analysis, t test (Mann‒Whitney test), ***p < 0.0001. **E** The Gluc activity of the supernatant and cell lysate was measured at the indicated time points. At 36 and 60 hpi, the supernatant and cell lysate were collected and used to monitor Gluc activity. The error bars indicate the standard deviations from three independent experiments.

The double-reporter FHV-1 was generated by homologous recombination between a donor plasmid containing a left arm, an mNeonGreen-F2A-Gluc expression cassette, a right arm, and the FHV-1 genome (GenBank accession no. PP942287, Figure 1A). Briefly, the double-reporter gene expression cassette was inserted into the middle of FHV-1 gG. To construct the homologous recombination donor plasmid (HRDP), the mNeonGreen-F2A-Gluc fragment was first synthesized by Wuhan Bozhongkang Biology Technology Company (China) to replace the EGFP-F2A-EGFP-T2A-EGFP of the PS531 plasmid [14]. Second, the left and right arms of FHV-1 were used to replace the left and right arms of PS531, respectively. The left arm covers nucleotides 117,379 to 118,850, and the right arm contains the fragment from 119,367 to 120,930 in the FHV-1 strain (GenBank no. MH070348.1).

To produce the double-reporter FHV-1, 4 μg of HRDP was transfected into CRFK cells; then, the transfected CRFK cells were infected with wild-type FHV-1 at 4 h post-infection (hpi). At 48 hpi, the supernatant was harvested and used as a sample to prepare the purified double reporter FHV-1 by isolating the mNeonGreen-positive plaques over four rounds via a plaque assay.

### PCR assay

To analyse the stability of the inserted gene in the double-reporter FHV-1, a PCR assay was carried out in which primer-F (5′-CAGCAAGATCCAGGGCCAGGT-3′) and primer-R (5′-TGGACCACCAATAAGATCAC-3′) were identified. Briefly, the genome of FHV-1 was extracted using the QIAamp MinElute Virus Spin Kit (57,704; Qiagen, Germany), and PCR was performed using the PrimeSTAR Max DNA Polymerase (R045A; TaKaRa, Japan). The PCR products were analysed by agarose gel electrophoresis.

### Time course of viral growth, Gluc activity, and mNeonGreen fluorescence

The CRFK cells were infected with wild-type FHV-1 or the double reporter FHV-1 at a multiplicity of infection (MOI) of 0.05. The supernatants were collected at 12, 24, 36, 48, 60, and 72 hpi. The viral titre and Gluc activity of each sample were subsequently determined using a plaque assay and a luciferase assay. In addition to the supernatant collection, the mNeonGreen fluorescence signal was measured using a Spark multifunctional microplate detector.

### Plaque assay

A plaque assay was carried out to purify the double-reporter FHV-1 and measure the viral titre via a previously reported method [14]. Briefly, 100 μL of the diluted viruses were added to each well of 6-well plates containing CRFK cells, and the plates were subsequently incubated at 37 °C with 5% CO_2_ for 1 h to allow the virus to enter the cells. Then, a first layer of agar was added to each well. After 48 hpi, a second layer of agar containing neutral red (71,028,260, Sinopharm Chemical Reagent, China) was added. The plaque number was determined after an additional 24 h of incubation. The viral titre was calculated as the plaque-forming units (PFU) per millilitre.

### Luciferase assay

The Gaussia luciferase assay was performed as described previously [23]. Briefly, 20 μL of supernatant or cell lysate was automatically mixed with 50 μL of substrate in a 96-well black plate, and the Gluc activity of each sample was measured using a Spark multifunctional microplate detector.

### Cell viability and antiviral assays

These assays were performed using previously described methods [24]. Before the antiviral assay, the cytotoxicity of ganciclovir toward CRFK cells was measured using a CCK8 kit. Briefly, CRFK cells were incubated for 24 h at 37 °C in 5% CO_2_ with the serially diluted drug ganciclovir at concentrations ranging from 0 to 100 μM. Then, 10 μL of CCK-8 solution was added to each well, and the optical density (OD) was measured using a Spark multifunctional microplate detector.

The antiviral assay was subsequently performed as described in a previous study [24]. The concentrations of all the drugs needed to be lower than the doses at which the viability of the CRFK cells remained at 80% or higher. Briefly, CRFK cells were infected with the double reporter FHV-1 at an MOI of 0.05 for 1 h, and then, the infected cells were separately incubated with the indicated concentrations of ganciclovir (0–100 μM) for 24 h at 37 °C in 5% CO_2_. At 60 hpi, the mNeonGreen signal value was monitored using a Spark multifunctional microplate detector, and the supernatant was harvested and used to determine the viral titre and Gluc activity. The 50% effective concentration (EC_50_) was calculated using nonlinear regression in GraphPad Prism software on the basis of the viral titre, Gluc activity, and mNeonGreen fluorescence value.

### Western blot

The assay was performed as previously described [14]. In brief, CRFK cells were infected with the double reporter FHV-1 at an MOI of 0.05 at 37 °C in 5% CO_2_. The cells were separately collected at 12, 24, 36, 48, and 60 hpi. Each cell lysate was subjected to SDS‒PAGE analysis, which was then followed by transfer to PVDF membranes (IPVH00010, Millipore, USA). The monoclonal antibody against mNeonGreen at a 1:3000 dilution and the goat anti-rabbit secondary antibody at a 1:3000 dilution were subsequently used to blot the mNeonGreen. The signal was measured using enhanced chemiluminescence (ECL) Western blotting reagent (34,580, Thermo Scientific, USA).

## Results

### Constructing a double-reporter FHV-1

To develop a rapid, sensitive, and convenient method for antiviral drug screening, we engineered two reporter genes, mNeonGreen and Gluc, to locate gG via homologous recombination (Figure 1A). mNeonGreen is a bright-green fluorescent protein [21]. Gluc is a secreted luciferase that can be released into cell culture media [22, 23]. These two reporter genes are connected by the F2A linker, which is located downstream of the strong promoter CAG and a rabbit β-globin intron. Our previous work revealed that the combination of the promoter CAG and the rabbit β-globin intron resulted in greater gene transcription than did the promoter CAG alone [14]. F2A was engineered to ensure that mNeonGreen and Gluc were properly processed. Compared with the wild-type FHV-1, the double-reporter FHV-1 has a smaller plaque size and can express fluorescent proteins (Figures 1B–D). Gluc activity can be detected in the supernatant and cell lysate at 36 and 60 hpi, and the Gluc activity of the sample at 36 hpi was higher than that at 60 hpi, while the supernatant had more Gluc than the cell lysate did (Figure 1E). These results indicate that the double-reporter FHV-1 was successfully generated.

### Analysing the stability of the double-reporter FHV-1 expression of mNeonGreen and Gluc

**Figure 2:**
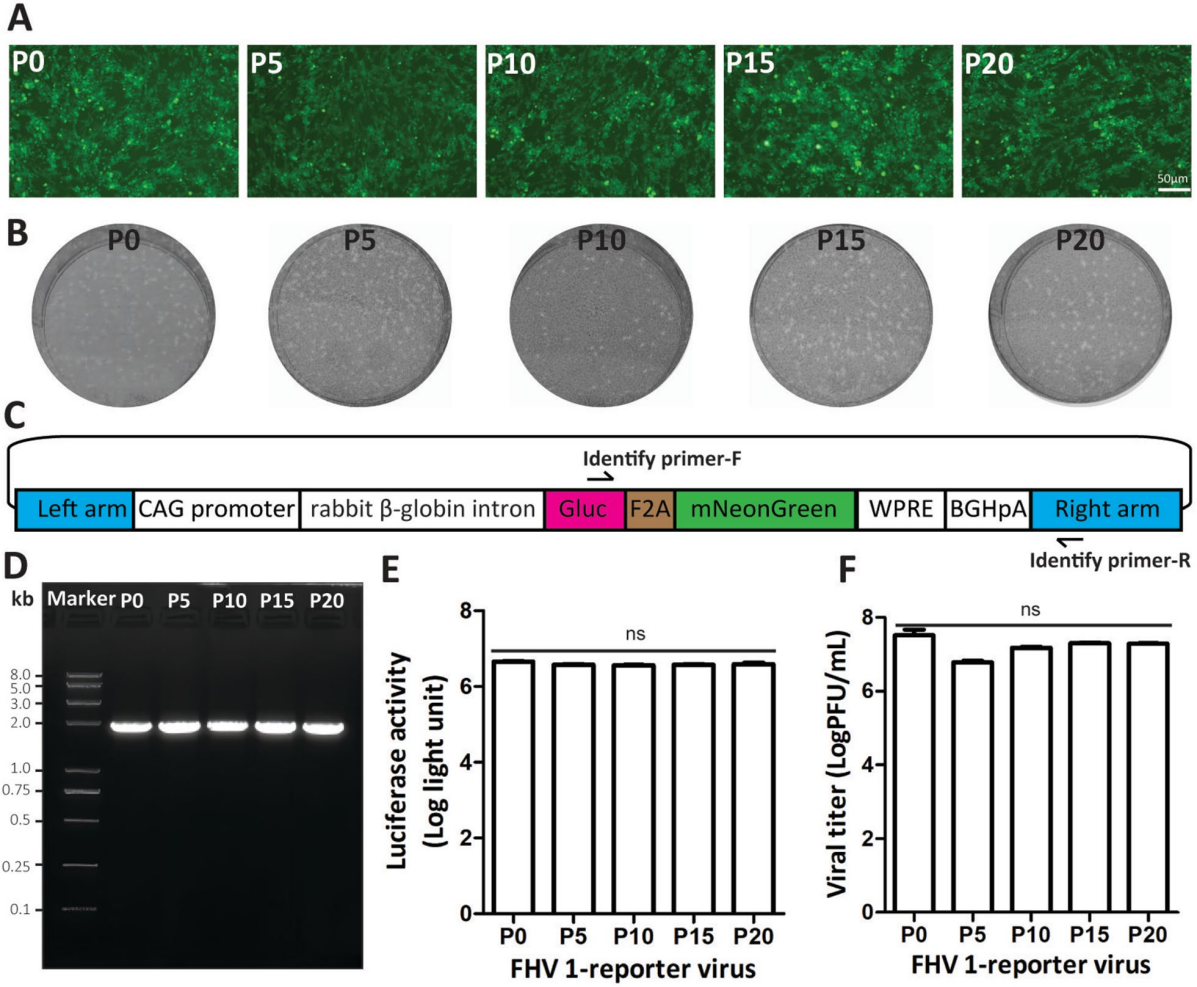
**Stability of the double-reporter FHV-1 in CRFK cells.** **A** Double-reporter viruses (P0, P5, P10, P15, and P20) can infect CRFK cells and express mNeonGreen. The fluorescent protein was observed by fluorescence microscopy at 48 hpi. **B** Plaque sizes of viruses P0, P5, P10, P15, and P20. **C** The specific PCR primers for amplifying the Gluc-2A-mNeonGreen of viruses P0, P5, P10, P15, and P20. The identified primer-F was located in the Gluc gene, and the identified primer-R was located in the right arm. **D** The PCR product of each virus was analysed by agarose gel electrophoresis. **E** Gluc activity of viruses P0, P5, P10, P15, and P20 infecting CRFK cells at an MOI of 0.005 for 48 h. Statistical analysis, one-way ANOVA (Kruskal‒Wallis test); ns: *p* > 0.05. **F** Viral titres of viruses P0, P5, P10, P15, and P20 infecting CRFK cells at an MOI of 0.05 for 48 h. Statistical analysis, one-way ANOVA (Kruskal‒Wallis test), ns: *p* > 0.05.

The stability of the double-reporter FHV-1 is a prerequisite for its application in antiviral drug screening. We analysed the stability of the reporter virus by monitoring the mNeonGreen fluorescence signal, plaque size, reporter gene, Gluc activity and viral titre. We continuously passaged the double-reporter FHV-1 for twenty passages in CRFK cells. Viruses from P0, P5, P10, P15, and P20 were used to test the stability. These five viruses can infect CRFK cells, express the green fluorescent protein mNeonGreen (Figure 2A), and produce similar plaque sizes (Figure 2B). A PCR assay was subsequently performed via primer-F and primer-R to target the fragment comprising Gluc and the right arm (Figure 2C). These five viruses produced a single band (Figure 2D), and the band length was consistent with the real length. Next, these five viruses were used to infect CRFK cells at an MOI of 0.05, and the supernatant was then collected at 48 hpi and used to measure the Gluc activity and viral titre. As shown in Figures 2E and F, these five viruses presented similar Gluc activity and viral titres. Collectively, these results indicate that the double-reporter FHV-1 is stable during passage in CRFK cells.

### Measuring the time curve of viral propagation, Gluc activity, and mNeonGreen expression

**Figure 3:**
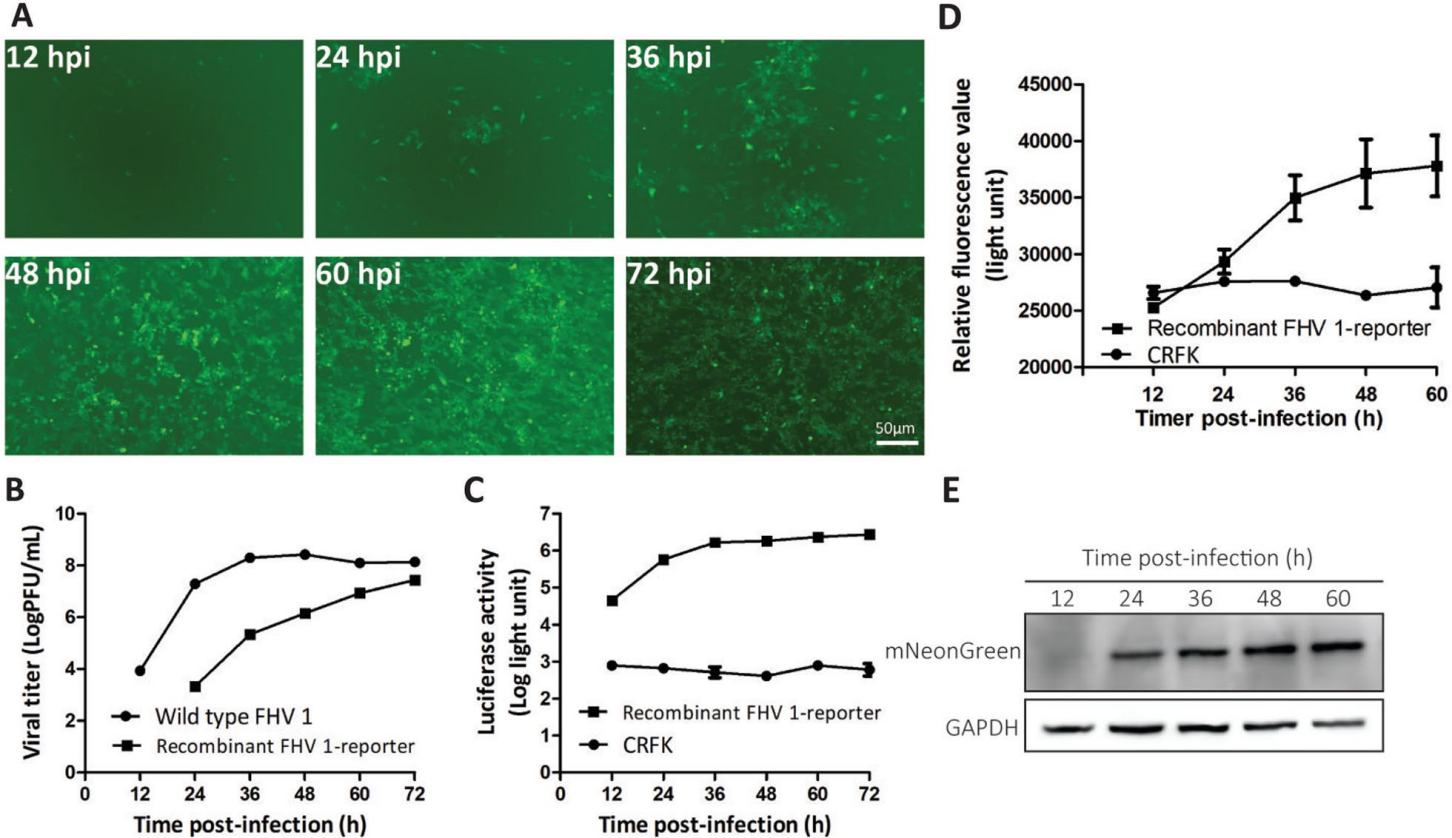
**Characteristics of the double-reporter FHV-1.** The CRFK cells were infected with the wild-type FHV-1 or the double-reporter FHV-1 at an MOI of 0.05. mNeonGreen fluorescence was observed, and the supernatant and cell lysate were collected at 12, 24, 36, 48, 60 and 72 hpi. **A** Time course of the number of mNeonGreen-positive cells at the indicated time points. **B** Viral growth curves of the wild type and the FHV-1 double reporter. **C** Time course of Gluc activity at the indicated time points. **D** Time course of the mNeonGreen fluorescence value at the indicated time points. **E** The time course of the mNeonGreen expression level was determined by Western blotting. The error bars indicate the standard deviations from three independent experiments.

In antiviral drug screening, it is necessary to establish time curves for viral propagation, Gluc activity, and mNeonGreen expression. The reporter virus and the wild-type virus were used to infect CRFK cells at an MOI of 0.05. As shown in Figures 3A and D, the mNeonGreen fluorescence signal increased with time. The Western blot also provided the same results as the fluorescent signals (Figure 3E). Additionally, the mNeonGreen fluorescence value determined via the reader was similar to that for the negative control CRFK, which indicates that the signal-to-background ratio was low. The growth curves of the reporter virus and the wild-type virus revealed that the reporter virus grew slower than the wild-type virus did (Figure 3B). The Gluc activity increased with increasing degree of viral replication, and the negative control CRFK had a low background value (Figure 3C). These results indicate that the suitable parameters for antiviral drug screening include (1) an infection dose of an MOI of 0.05 and (2) the time for mNeonGreen observation, Gluc activity measurement, and viral titre determination at 48 hpi.

### Evaluating the antiviral ability of the FHV-1 double reporter

**Figure 4:**
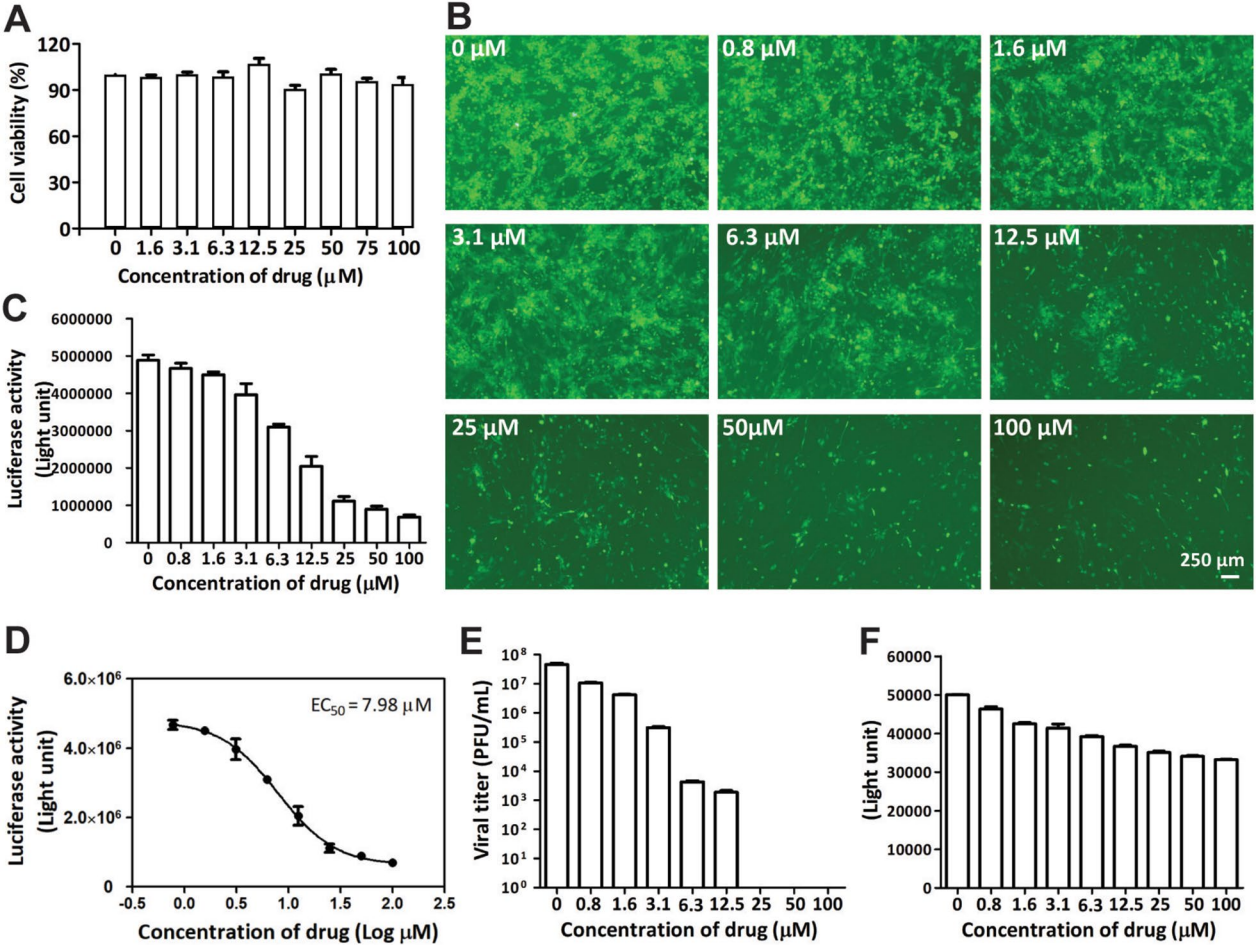
**Antiviral analysis of ganciclovir on the basis of the viral titre, Gluc activity, and mNeonGreen fluorescence. A** The cytotoxicity of ganciclovir was determined on the basis of cell viability via the CCK-8 method. Ganciclovir was serially diluted to concentrations ranging from 0 to 100 μM and added to CRFK cells. The error bars indicate the standard deviations from three independent experiments. **B**–**F** Antiviral analysis of ganciclovir. The CRFK cells were infected with the double reporter FHV-1 and then treated with serially diluted ganciclovir from 0 to 100 μM. The mNeonGreen-positive cells were observed via a fluorescence microscope (**B**). The activity (**C**) and EC_50_ (**D**) values of Gluc were determined via a luciferase assay. Moreover, the viral titre (**E**) and mNeonGreen fluorescence (**F**) were determined via a plaque assay and a Spark multifunctional microplate detector, respectively. The error bars indicate the standard deviations from three independent experiments.

Next, we used ganciclovir to evaluate the ability of the double-reporter virus for antiviral drug screening. Previous studies have shown that this drug can inhibit FHV-1 or HSV-1 [10]. First, we analysed its cytotoxicity in CRFK cells via the CCK8 method. The concentration of ganciclovir used to maintain cell viability above 90% was 100 μM (Figure 4A). Therefore, we determined that the highest concentration of ganciclovir for the antiviral assay was 100 μM.

The CRFK cells were infected with the double reporter FHV-1 at an MOI of 0.05 and treated with different concentrations of ganciclovir (0–100 μM). The viral titre, Gluc activity, and mNeonGreen signal were subsequently measured. We found that ganciclovir was efficacious against FHV-1 on the basis of the number of mNeonGreen-positive cells (Figure 4B), Gluc activity (Figures 4C and D), viral titre (Figure 4E), and mNeonGreen fluorescence signal value (Figure 4F). The EC_50_ of ganciclovir was 7.98 μM (Gluc assay, Figure 4D), which is consistent with previous studies [10]. Collectively, these results demonstrate that the double reporter FHV-1 is a rapid, sensitive, and convenient tool for antiviral drug discovery.

## Discussion

Overall, we successfully developed a double-reporter FHV-1 that can be used to rapidly, sensitively, and conveniently screen drugs for combating FHV-1. Previously, drugs for treating FHV-1 disease in felines were usually based on the PRNT assay in vitro. This method is efficient but time consuming and laborious. Before our work, there was no established engineered double-reporter FHV-1 for antiviral screening assays. Our tool has mNeonGreen and Gluc reporter genes, which can be used to analyse the efficiency of drugs against FHV-1 by directly using a fluorescence microscope to visualize the expression level of mNeonGreen and by directly measuring the luciferase activity of the supernatant, without cell lysis, to assess the inhibitory ability of drugs against FHV-1. However, we found that the fluorescence of mNeonGreen was slightly greater than that of the CRFK background (Figure 3D) and produced a low signal-to-background ratio. The Western blot results confirmed that the expression of mNeonGreen increased with time (Figure 3E), which correlated well with viral replication (Figure 3B). The EC_50_ value of ganciclovir is 7.98 μM according to the Gluc assay (Figure 4D).

Once FHV-1 enters cells, a series of subsequent events, including transcription, replication, and expression (viral genes and inserted genes), occur. Then, the cytopathic effect (CPE) was observed. The CPE-based viral titre reflects the terminal result of viral propagation but cannot reflect the process of the viral lifecycle, whereas the Gluc activity reflects viral transcription, replication, and translation [25–28]. Even if no CPE is formed after the virus infects cells, the processes of viral transcription, replication, and translation are programmed to occur. Therefore, we observed that no virus was detected by the plaque assay at 25 μM, 50 μM, or 100 μM ganciclovir (Figure 4E), while the Gluc activity (Figures 4C and D) and mNeonGreen value (Figure 4F) could be measured, which indicates that the Gluc-based method is more sensitive and suitable for evaluating the inhibitory efficiency of drugs against FHV-1. Collectively, researchers can select mNeonGreen or Gluc (or a combination of them) as measurable targets for drug screening according to their aims at different stages of antiviral drug development.

In our study, we selected the drug ganciclovir, which was reported to be effective against FHV-1 in previous studies, to assess its antiviral ability via the use of a double-reporter virus. It can interfere with viral replication after a virus enters a cell [10]. The results showed that ganciclovir is efficacious. It has an EC_50_ value of 7.98 μM according to Gluc activity-based methods, which is consistent with a previous study [10]. This result clearly shows that this technique has potential for the treatment of feline FHV-1 disease. In fact, Ledbetter EC and coworkers confirmed that ganciclovir and famciclovir can reduce clinical ocular disease scores and corneal inflammation in vivo [29]. In our other project, we plan to analyse the prevalence of FHV-1 on the basis of the antibody against FHV-1 from felines in China. More than 100 serum samples from felines have been collected from different veterinary hospitals in China. Now, we are testing the antibody against FHV-1 from these samples on the basis of the double reporter FHV-1, and the results confirm that the reporter virus is effective for screening entry inhibitors against FHV-1 (data not shown). In the future, we will use the FHV-1 reporter to screen more drugs and compounds with different mechanisms against FHV-1.

Overall, this tool is effective for screening antiviral drugs and will facilitate the discovery of drugs against FHV-1.

## Data Availability

The data that support the findings of this study are available from the corresponding author upon reasonable request.
